# Senescence Mediated by p16^INK4a^ Impedes Reprogramming of Human Corneal Endothelial Cells into Neural Crest Progenitors

**DOI:** 10.1038/srep35166

**Published:** 2016-10-14

**Authors:** Wen-Juan Lu, Scheffer C. G. Tseng, Shuangling Chen, Sean Tighe, Yuan Zhang, Xin Liu, Szu-Yu Chen, Chen-Wei Su, Ying-Ting Zhu

**Affiliations:** 1 Department of Ophthalmology, Ninth People’s Hospital, Shanghai Jiao Tong University School of Medicine, Shanghai, China; 2 R&D Department, Tissue Tech, Inc., Ocular Surface Research & Education Foundation, Miami, FL, USA; 3 Ocular Surface Center, Ocular Surface Research & Education Foundation, Miami, FL, USA; 4 Dalian Central Hospital, Dalian City, Liaoning Province, China; 5 Department of Ophthalmology, Union Hospital, Tongji Medical College, Huazhong University of Science and Technology, Wuhan, China

## Abstract

Human corneal endothelial cells (HCECs) have limited proliferative capacity due to “contact-inhibition” at G1 phase. Such contact-inhibition can be delayed from Day 21 to Day 42 by switching EGF-containing SHEM to LIF/bFGF-containing MESCM through transient activation of LIF-JAK1-STAT3 signaling that delays eventual nuclear translocation of p16^INK4a^. Using the latter system, we have reported a novel tissue engineering technique by implementing 5 weekly knockdowns with p120 catenin (p120) and Kaiso siRNAs since Day 7 to achieve effective expansion of HCEC monolayers to a transplantable size with a normal HCEC density, through reprogramming of HCECs into neural crest progenitors by activating p120-Kaiso-RhoA-ROCK-canonical BMP signaling. Herein, we noted that a single knockdown with p120-Kaiso siRNAs at Day 42 failed to achieve such reprogramming when contact inhibition transitioned to senescence with nuclear translocation of p16^INK4a^. In contrast, 5 weekly knockdowns with p120-Kaiso siRNAs since Day 7 precluded senescence mediated by p16^INK4a^ by inducing nuclear translocation of Bmi1 because of sustained activation of JAK2-STAT3 signaling downstream of p120-Kaiso-RhoA-ROCK signaling. STAT3 or Bmi1 siRNA impeded nuclear exclusion of p16^INK4a^ and suppressed the reprogramming induced by p120-Kaiso siRNAs, suggesting that another important engineering strategy of HCEC lies in prevention of senescence mediated by nuclear translocation of p16^INK4a^.

HCECs play a pivotal role in regulating corneal stromal hydration and hence transparency by exerting effective barrier and pump functions (reviewed in refs 1,2). Unlike other species, HCECs are notorious for having limited proliferative capacity *in vivo*^3^ because of the mitotic block at the G1 phase of the cell cycle due to “contact-inhibition”^4^. Although there is a *bona fide* mitotic block of HCECs *in vivo,* tangential evidence has emerged suggesting the presence of HCEC progenitors in the corneal periphery or the junction between the corneal endothelium and the trabecular meshwork based on appearance of BrdU labeling^5^, expression of stem cell markers such as Oct3/4 and Wnt1^6^ following wounding, and formation of neurospheres with expression of nestin by EDTA-dissociated HCECs^7^,^8^,^9^,^10^. For the first time, our study has disclosed the feasibility of promoting proliferation of HCEC monolayers by reprogramming adult HCECs into neural-crest (NC) progenitors as evidenced by upregulation of ESC markers such as Nanog, Nestin, Oct4, SOX-2 and SSEA4 as well as NC markers such as AP2β, FOXD3 and SOX9, and nuclear translocation of Oct4, Sox2 and Nanog^11^. Such reprogramming depended on the activation of canonical BMP signaling^11^.

Previously, we have reported that mitotic block of HCECs due to contact inhibition is established after 3 weeks of culture in an EGF- and serum-containing medium termed SHEM^12^. However, a switch from SHEM to a serum-free LIF-containing medium termed MESCM delays contact inhibition by 3 weeks but nonetheless is insufficient to result in effective expansion of HCECs^13^. In contrast, in the latter system with MESCM, we have reported a novel tissue engineering technique by implementing 5 weekly knockdowns with p120 catenin (p120) and Kaiso siRNAs since Day 7 to achieve effective expansion of HCEC monolayers to a transplantable size with a normal HCEC density from 1/8 of a human cornea^11^ This successful outcome is correlated with reprogramming of HCECs into neural crest progenitors through selective activation of p120-Kaiso signaling coupled with activation of Rho-ROCK-canonical BMP signaling by p120-Kaiso knockdown^11^.

The aforementioned delay in contact inhibition is achieved by transit activation of LIF-JAK1-STAT3 signaling that also delays eventual nuclear translocation of p16^INK4a^ 13. p16^INK4a^ belongs to the family of cyclin-dependent kinase inhibitors involved in cell cycle arrest at the G1 phase^14^. Nuclear p16^INK4a^ is a hallmark of contact inhibition because p16^INK4a^ binds to CDK4/6 inhibiting its kinase activity thereby preventing Rb phosphorylation during G1 to S transition (reviewed in ref. 15). Because nuclear translocation of phosphorylated p16^INK4a^ leads to an irreversible arrest during the G1 transition of the cell cycle^16^,^17^,^18^, and p16INK4a has been identified as a marker for HCEC senescence^19^, we would like to test the hypothesis that overcoming of contact inhibition-induced senescence mediated by p16^INK4a^ is a pre-requisite for successful reprogramming of HCECs into human corneal endothelial progenitors.

## Results

### Nuclear p16^INK4a^ is a hallmark of contact inhibition of HCECs in MESCM

**Figure 1:**
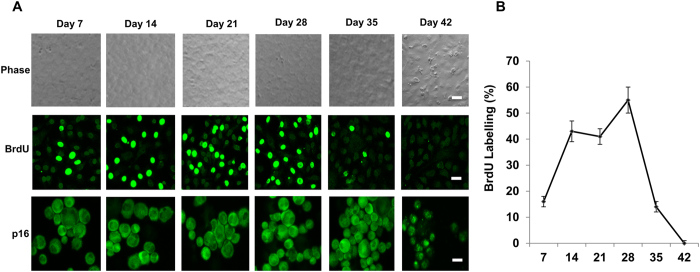
Nuclear translocation of p16^INK4a^ during contact inhibition in MESCM. HCECs cultured in MESCM for up to Day 42 were monitored by phase contrast microscopy (**A**, Scale bars: 25 μm), immunostaining of p16^INK4a^ (p16) (A, Scale bars: 25 μm), and BrdU labeling 24 h before termination (**A,B**).

Unlike the corneal endothelial cells of other species, such as rabbit and bovine, human corneal endothelial cells (HCECs) are notorious for their limited proliferative capacity *in vivo*^3^ because of the mitotic block at the G1 phase of the cell cycle due to “contact-inhibition”^4^. In our model system, contact inhibition is established at Day 21 when cultured in SHEM^12^, but delayed until Day 42 if cultured in MESCM, an event that is correlated with nuclear exclusion of p16^INK4a^ 13. We thus relooked the same model system in MESCM and noted that BrdU labeling was indeed abundant with as much as 57% BrdU positive cells between Day 21 to Day 28 in HCECs before Day 28 and gradually declined to a complete halt at Day 42 (Fig. 1). This result suggested that contact inhibition in HCECs cultured in MESCM was indeed re-established at Day 42 when p16^INK4a^ first appeared in the nucleus (Fig. 1), indicative of that nuclear p16^INK4a^ is a marker of contact inhibition of HCECs in this model system.

### Contact inhibition mediated by p16^INK4a^ leads to senescence

**Figure 2:**
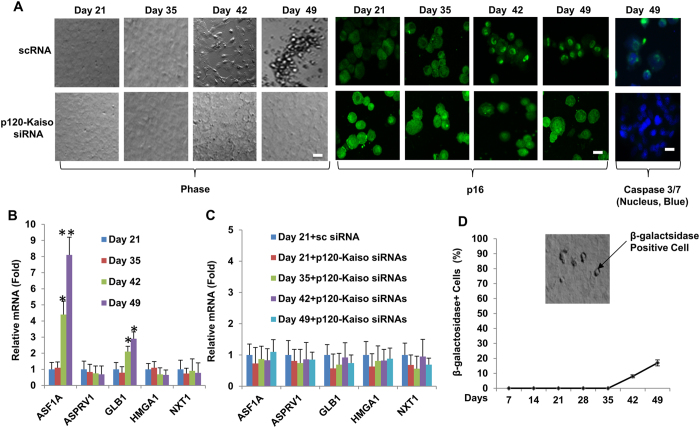
Prohibition of senescence and contact inhibition mediated by p16^INK4a^ by weekly knockdown of p120-Kaiso siRNAs from Day 7. HCECs cultured in MESCM up to Day 49 were monitored by phase contrast and cytolocalization of p16^INK4a^ by immunostaining (**A**). Cell apoptosis was demonstrated by green fluorescence using CellEvent^TM^ Caspase-3/7 Green Detection Reagent with nuclear counterstaining with Hoechst 33342 on Day 49 (A, Scale bars: 25 μm). The expression of senescence markers was measured by qRT-PCR [n = 3, *p < 0.05 and **p < 0.01, compared to the control without treatment (**B**) or with scrambled RNA (scRNA) at day 21 (**C**)]. The percentage of senescent cells were determined by β-galactosidase staining (**D**).

Because nuclear p16^INK4a^ is linked to senescence (reviewed by ref. 15 and is a marker for HCEC senescence^19^. we wondered whether the aforementioned contact inhibition also led to senescence of HCECs. Morphologically, the cells became flattened at Day 42 and floating at Day 49 (Fig. 2A), suggesting an “end replication” and an “end of cell life”^20^. During the period from Day 42 to Day 49 after the establishment of contact-inhibition in HCECs, there was continuous nuclear presence of p16^INK4a^ (Fig. 2A) and gradual upregulation of senescence markers such as ASF1A and GLB1 (Fig. 2B). At Day 42, the aforementioned morphological change was correlated with appearance of apoptotic marker caspase 3/7 (Fig. 2A) and 8% increase of percentage of senescent cells. In contrast, weekly knockdowns of p120-Kaiso siRNAs from Day 7 maintained the normal HCEC hexagonal morphology and density till Day 49 (Fig. 2A,C) as reported^11^ and prohibited nuclear translocation of p16^INK4a^ (Fig. 2A), expression of senescence markers (Fig. 2B), appearance of apoptotic marker caspase 3/7 (Fig. 2A) and increase of senescent cells (Fig. 2D, by 8% at Day 42 and 17% at Day 49 as a graduate increase of senescence was expected) during the entire experimental period. These results strongly suggest that weekly knockdown of p120-Kaiso prohibits p16^INK4a^-mediated contact inhibition and senescence.

### Reprogramming of HCECs by weekly knockdowns but not a single knockdown of p120-Kaiso at Day 42

**Figure 3:**
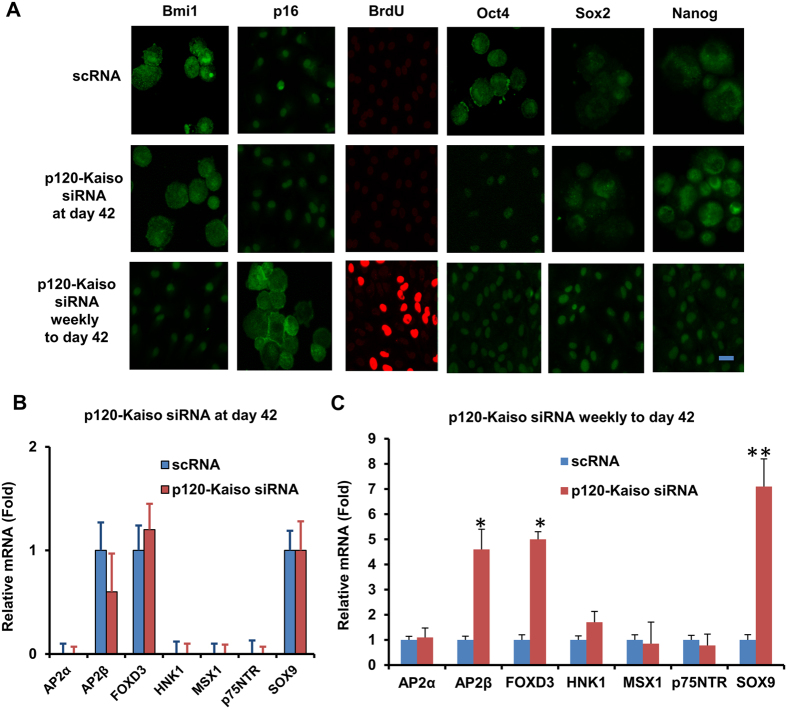
Reprogramming of HCECs to progenitors by weekly but not by single knockdown with p120-Kaiso siRNAs. HCECs received 5 weekly knockdowns since Day 7 or single knockdown at Day 42 with p120-Kaiso siRNAs or scRNA in MESCM. Immunostaining to Bmi1 and p16^INK4a^ was correlated with BrdU labeling and immunostaining to Oct4, Sox2 and Nonog (**A**, Scale bars: 25 μm). Expression of neural crest markers analyzed at Day 42 was measured by qRT-PCR after a single knockdown at Day 42 (**B**) or weekly knockdowns from Day 7 till Day 42 (C, n = 3, *p < 0.05 and **p < 0.01, compared to scRNA control).

We have recently reported that weekly p120-Kaiso knockdown since Day 7 maintained the aforementioned proliferation of HCEC monolayers by reprogramming these cells into neural crest–like progenitors. Such reprogramming is characterized by an increase of BrdU labeling, up-regulation of ESC markers such as Nanog, Nestin, Oct4, SOX-2, and SSEA4 as well as neural crest markers such as AP2β, FOXD3, and SOX9, and activation of miR302b-Oct4-Sox2-Nanog network via canonical BMP signaling^11^. Consistent with what we have reported^11^, such reprogramming indeed ensued in HCECs receiving 5 weekly knockdowns of p120-Kaiso since Day 7 as evidenced by positive BrdU labeling, nuclear Oct4, Sox2 and Nanog (Fig. 3A), and upregulation of all NC markers such as AP2β, FOXD3, and SOX9 in MESCM (Fig. 3C). In contrast, a single knockdown of p120-Kaiso siRNAs at Day 42 could not achieved such reprogramming. There was neither BrdU labeling (Fig. 3A) nor expression of NC markers (Fig. 3B). There was nuclear translocation of Oct4 but not Sox2 or Nanog (Fig. 3A). Because p16^INK4a^ is negatively mediated by Bmi1^21^,^22^,^23^, a key regulatory component of Polycomb repressive complex 1 complex (PRC1)^24^, we then looked into cytolocalization of p16^INK4a^ and Bmi1. At Day 42, p16^INK4a^ was still in the nucleus while Bmi1 in the cytoplasm (Fig. 3A) when a single knockdown of p120-Kaiso siRNAs was carried out. In contrast, p16^INK4a^ was in the cytoplasm while Bmi1 in the nucleus (Fig. 3A) for HCECs receiving weekly knockdowns since Day 7. In addition, HCECs at Day 42 in MESCM yielded few surviving cells that could not be expanded after passaging (not shown). Collectively, these results suggested that reprogramming of HCECs to their progenitors induced by p120-Kaiso siRNAs requires nuclear exclusion of p16^INK4a^, which is coupled by nuclear translocation of Bmi1.

### Balancing action between p16^INK4a^ and Bmi1

**Figure 4:**
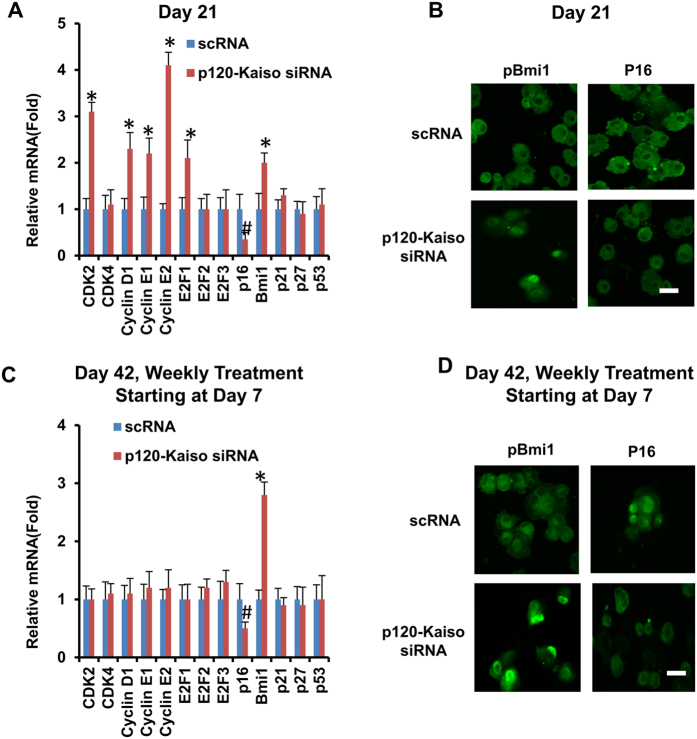
Balancing action between p16^INK4a^ and Bmi1. HCECs received single knockdown at Day 21 (**A,B**) or 5 weekly knockdowns by scRNA or p120-Kaiso siRNAs since Day 7 (**C,D**) in MESCM. Expression of various cell cycle regulators was measured by qRT-PCR (A and C, *p < 0.05 and ^#^p < 0.05 when compared to scRNA control). Cytolocalization of Bmi1 and p16^INK4a^ was assessed by immunostaining (B and D, Scale bars: 25 μm).

Because Bmi1 is a key negative regulator of p16^INK4a^ 21,22,23, we then asked whether Bmi1 played a major role in mediating expression and nuclear translocation of p16^INK4a^ during the reprogramming process induced by p120-Kaiso siRNAs. We first examined expression of a number of cell cycle regulators in HCEC receiving weekly knockdown by p120-Kaiso siRNAs up to Day 21, when contact inhibition remains to be precluded in MESCM. qRT-PCR revealed upregulation of a number of positive cell cycle genes such as CDK2, cyclin D1, cyclin E1, cyclin E2, E2F1 and Bmi1 (Fig. 4A). Interestingly, there was notable downregulation of p16^INK4a^ transcript but upregulation of Bmi1 (Fig. 4A). Immunostaining confirmed cytoplasmic location of p16^INK4a^ but nuclear localization of Bmi1 (Fig. 4B). The same results were held up by Day 42 following weekly knockdown of p120-Kaiso, resulting in unique downregulation of p16^INK4a^ transcript but upregulation of Bmi1 transcript (Fig. 4C). This finding was correlated with their anticipated respective cytolocalization of p16^INK4a^ and Bmi1 (Fig. 4D). Collectively, these data disclosed a strong counterbalancing action exerted by Bmi1 toward p16^INK4a^.

### Bmi1 siRNA mitigates reprogramming of HCECs to progenitors

**Figure 5:**
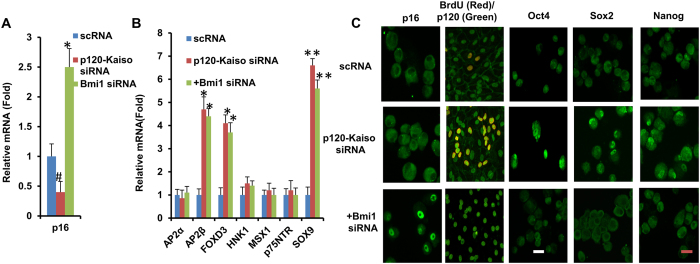
Mitigation of reprogramming of HCECs to progenitors by p120-Kaiso knockdown by Bmi1 siRNA. At Day 35, Bmi1 siRNA was added to HCECs receiving 5 weekly knockdowns with scRNA or p120-Kaiso siRNAs since Day 7. Transcript expression of p16^INK4a^ (p16) (**A**) and various neural crest markers was compared by qRT-PCR (B, n = 3, ^#^p < 0.05, *p < 0.05 and **p < 0.01 compared to scRNA control). Cytolocalization of p16^INK4a^, Oct4, Sox2, Nanog, and BrdU (red)/p120 (green) was also compared by immunostaining (**C**, Scale bars: 25 μm).

To substantiate a causative relationship between Bmi1 and p16^INK4a^ in achieving the reprogramming of HCECs to progenitors induced by p120-Kaiso siRNAs (Fig. 4), we performed Bmi1 siRNA knockdown on Day 35 when the 5^th^ p120-Kaiso knockdown was administered. As expected, Bmi1 siRNA upregulated expression of p16^INK4a^ transcript that was downregulated by p120-Kaiso siRNAs (Fig. 5A), and induced nuclear translocation of p16^INK4a^ (Fig. 5C). Consequently, the salient features of the said reprogramming of HCECs were also halted by Bmi1 siRNA as evidenced by the loss of BrdU labeling and nuclear translocation of Oct 4, Sox2 and Nanog (Fig. 5C). Bmi1 siRNA did not alter transcript upregulation of NC markers, AP2β, FOXD3, and SOX9 (Fig. 5B), suggesting that Bmi1 signaling was not linked to transcriptional control of the over-expression of NC markers. Because Bmi1 siRNA also did not alter nuclear localization of p120 (Fig. 5C) and nuclear exclusion of Kaiso (not shown) that have been reported under knockdown by p120-Kaiso siRNAs^11^,^25^, it also suggests that Bmi1 signaling is not linked to p120-Kaiso signaling triggered by p120-Kaiso knockdown.

### JAK2-STAT3 signaling as a downstream of RhoA-ROCK signaling leading to nuclear Bmi1

**Figure 6:**
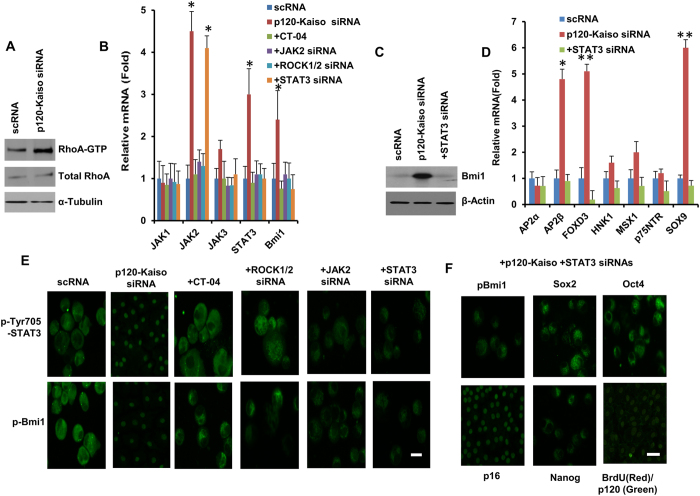
An important role of JAK2-STAT3 signaling in eliciting nuclear Bmi1. HCECs received 5 weekly knockdowns with scRNA or p120-Kaiso siRNAs starting at Day 7. On Day 35, Rho inhibitor CT-04, ROCK1/2 siRNA, JAK2 siRNA or STAT3 siRNA were added in addition to the p120-Kaiso siRNA treatment. Activation of RhoA was measured by Western blotting normalized by α-tubulin (**A**). Activation of JAK2-STAT3-Bmi1 signaling coupled with Rho-ROCK signaling was shown by qRT-PCR (**B,D**), Western blotting normalized by β-actin (**C**) and immunostaining to various markers (**E,F**, Scale bars: 25 μm).

We have reported that activation of RhoA by p120-Kaiso knockdown is required to elicit RhoA-ROCK-canonical BMP signaling in order to unlock mitotic block by reprogramming adult HCECs to their progenitors in LIF-containing MESCM^11^. We have also noted that without p120-Kaiso knockdown, LIF delays contact inhibition of HCEC monolayer by 3 weeks, probably through a transit activation of LIF-JAK1-STAT3 signaling to inhibit nuclear translocation of p16^INK4a^ without leading to the said reprogramming^13^. In contrast, 5 weekly knockdown of p120-Kaiso since Day 7 dramatically activated not only RhoA (Fig. 6A) as reported^11^ but also promoted a sustained transcript expression of JAK2 and STAT3 (Fig. 6B). Because Bmi1 is a downstream mediator of STAT3 but an upstream regulator of p16^INK4a^ and a known signaling for reprogramming (reviewed in ref. 26), we studied the role of Bmi1 in our reprogramming model. As expected, transcript and protein expression of Bmi1 signaling were upregulated by p120-Kaiso siRNAs (Fig. 6B,C). Such an upregulation was inhibited by JAK2 or STAT3 siRNA (Fig. 6B,C), suggesting that Bmi1 is downstream of JAK2 and STAT3. In addition, STAT3 siRNA also attenuated nuclear staining of pBmi1 (Fig. 6E). Interestingly, such blockade of nuclear pBmi1 by STAT3 siRNA was correlated to nuclear localization of p16^INK4a^ (Fig. 6F), suggesting that nuclear Bmi1 is indeed required for inhibiting nuclear p16^INK4a^ in order to allow the process of the reprogramming induced by p120-Kaiso siRNAs. Because downregulation of STAT3 by its siRNA also attenuated transcript expression of NC markers such as AP2β, FOXD3 and SOX9, BrdU labeling and nuclear translocation of Oct4, Sox2 and Nanog (Fig. 6D,G). Such JAK2-STAT3 signaling was probably downstream of RhoA-ROCK signaling because Rho inhibitor CT-04 and ROCK1/2 siRNAs inhibited mRNA expression of JAK2 and STAT3 (Fig. 6B) and blocked nuclear translocation of pSTAT3 Tyr 705 (Fig. 6D). Therefore, preclusion of nuclear p16^INK4a^ by nuclear translocation of Bmi1 through sustained activation of JAK2-STAT3 signaling, which served as the downstream of p120-Kaiso-RhoA-ROCK signaling, was required for the said reprogramming.

## Discussion

In our model system of HCECs, contact inhibition is re-established in 3 weeks when cultured in SHEM^12^ and delayed in 6 weeks when cultured in MESCM^13^. Herein, we noted that such re-establishment of contact inhibition in MESCM was correlated with nuclear localization of p16^INK4a^ (Fig. 1). Because p16^INK4a^ regulates G1-S transition^27^, our finding further elucidated a pivotal role of p16^INK4a^ in governing contact inhibition of HCECs, which has been recognized to occur at the G1 phase of the cell cycle^4^. Our study further showed that such irreversible mitotic arrest at G1 phase eventually led to senescence as evidenced by cell detachment from the culture dishes, expression of apoptotic marker caspase 3/7, positive staining of senescence marker β-galactosidase, and over-expression of senescence markers such as ASF1A and GLB1 (Fig. 2).

Several studies have shown that senescence is mediated by up-regulation of cyclin-dependent kinase inhibitors such as p16^INK4a^, p21^CIP1^, p27^KIP1^ or p53^14^ *in vivo, ex vivo* and *in vitro*^4^,^28^,^29^. Expression of p16^INK4a^ but not p21^CIP1^, p27^KIP1^ and p53, was remarkably elevated in the contact-inhibited HCECs from old donors (>50 years old) compared to that from young donors (<30 years old)^29^. Interestingly, an age-dependent increase of senescence-associated GLB1 activity in contact-inhibited in *ex vivo* HCECs was only noted in central but not peripheral HCECs^19^. Intriguingly, we have only succeeded in reprogramming peripheral, but not central corneal endothelial cells into progenitors (not shown) probably due to low replication competence in central corneal endothelial cells. Re-establishment of contact inhibition and senescence was a barrier against p120-Kaiso siRNA’s ability to further reprogram HCECs into neural crest progenitors. This notion was supported by the lack of BrdU labeling, expression of NC markers, and nuclear translocation of Sox2 and Nanog when a single knockdown by p120-Kaiso siRNAs was performed at Day 42 (Fig. 3). This finding also bears resemblance of what has been reported that senescence is triggered through upregulation of p16^INK4a^, p21^CIP1^ and p53 by H-RAS^G12V^ (which leads to elimination of the intrinsic GTPase activity) and is a barrier against successful reprogramming of iPSCs by over-expression of Oct4, Sox2, Klf4 and c-Myc in IMR90 human fibroblasts^14^,^30^. In contrast, consistent with our previous report^11^, reprogramming of HCECs to progenitors was indeed successful if weekly knockdown by p120-Kaiso siRNAs was carried out since Day 7 till Day 42 as evidenced by BrdU labeling and expression of NC markers and nuclear staining of Oct4, Sox2 and Nanog (Fig. 3). The lack of senescence was evidenced by downregulation of ASF1A and GLB1, the lack of expression of apoptotic marker caspase 3/7, and nuclear exclusion of p16^INK4a^ (Fig. 2). Hence, we conclude that the nuclear translocation of p16^INK4a^ leads to contact inhibition and subsequent senescence that constitute as a barrier against the said reprogramming.

In fact, others have reported that senescence coupled with apoptosis is a barrier against the acquisition of ESC-like proliferation during reprogramming of adult somatic cells to iPSCs^31^,^32^,^33^,^34^, which involves three distinct phases: (1) initiation characterized by mesenchymal-to-epithelial transition (MET), (2) maturation characterized by expression of Yamanaka factors, and (3) stabilization characterized by DNA methylation and histone modification (reviewed in ref. 35). It should be noted that reprogramming of adult HCEC to neural crest progenitors does not progress all the way to iPSCs. It involves upregulation of three of Yamanaka factors such as Nanog, Oct4 and Sox-2 with nuclear translocation, NC markers, and miRNA 302b/c that fosters expression of the aforementioned ESC markers. Such reprogramming leads to enhanced BrdU labeling and successful expansion of HCEC monolayers^11^. It remains to be determined whether DNA methylation and histone modification actually occur in the reprogramming of HCEC to neural crest progenitors. It should be also noted that the said reprogramming is not related to endothelial-mesenchymal transition (EMT). We have reported that EMT occurs only if cell-cell junctions of HCECs are disrupted by EDTA with or without trypsin and is mediated by activation of canonical Wnt signaling^11^. In contrast, EMT does not occur in the current model where HCECs are isolated by collagenase digestion (without EDTA ± trypsin) to preserve cell-cell junctions and cell-matrix interaction^24^. The latter method does not activate canonical Wnt signaling with or without p120-Kaiso knockdown and therefore does not cause EMT^11^. This notion is further supported by our finding that resultant HCEC monolayers retained the hexagonal shape, small in size and normal cell density (Fig. 2A). Collectively, we conclude that this novel engineering strategy based on selective activation of p120-Kaiso-BMP signaling leads to reprogramming of HCEC into NC progrogenitors is linked to suppression of EMT and suppression of senescence.

Bmi1 as a key regulatory component of Polycomb repressive complex 1 complex (PRC1)^24^ is necessary for the self-renewal of normal hematopoietic stem cells^36^ and neural stem cells^23^. Because p16^INK4a^ is negatively regulated by Bmi1^21^,^22^,^23^, we further explored the action mechanism by delineating a causative relationship between p16^INK4a^ and Bmi1 in reprogramming achieved by weekly knockdown with p120-Kaiso siRNAs. We first noted downregulation of p16^INK4a^ transcript and upregulation of Bmi1 mRNA in conjunction with upregulation of cell cycle regulators such as CDK2, cyclin D1 cyclin E1, cyclin E2 and E2F1 at Day 21 (Fig. 4A). However, by Day 42 when contact inhibition and senescence occurred, expression of the Bmi1 transcript was upregulated while that of the p16^INK4a^ transcript without aforementioned changes of cell cycle regulators (Fig. 4C), suggesting the close relationship between p16^INK4a^ and Bmi1 in creating the said reprogramming barrier mediated by contact inhibition and senescence. We then noted that Bmi1 siRNA upregulated expression of p16^INK4a^ transcript as expected on Day 35, but also induced nuclear staining of p16^INK4a^ along with blockade of BrdU labeling and attenuation of nuclear translocation of Oct 4, Sox2 and Nanog (Fig. 5). Interestingly, knockdown of p16 did not prohibit nuclear translocation of p16^INK4a^ yet attenuated its immunostaining and cause significant inhibition of senescence (not shown), suggesting that Bmi1 but not p16^INK4a^ controls nuclear translocation of p16^INK4a^ leading to senescence. These findings collectively allow us to conclude that upregulation of Bmi1 is critical to downregulate p16^INK4a^ so as to delay contact inhibition and allow the said reprogramming by p120-Kaiso siRNAs. Because Bmi1 siRNA did not affect the overexpression of NC markers (Fig. 5B), we speculate that the signaling controlling expression of ESC markers differs from that controlling expression of NC markers. Beside interaction with p16^INK4a^, it remains unclear whether Bmi-1 in our model system may also interact with Wnt and Notch signaling^37^, Akt signaling^38^,^39^, and Hedgehog signaling^40^ as reported in others.

One upstream mediator of Bmi1 is STAT3, a known signaling for reprogramming (reviewed in ref. 26), which may be regulated by RhoA^41^ through JAK2-STAT3 signaling^42^, and by ROCK1 through JAK2-STAT3 pathway (reviewed in refs 43,44). We have reported that the said reprogramming by p120-Kaiso siRNAs involves the activation of RhoA-ROCK-canonical BMP signaling^11^. Although MESCM contains LIF, we noted that LIF-JAK1-STAT3 signaling alone, i.e., without p120-Kaiso siRNAs, does not reprogram HCECs into progenitors^13^. Herein, we noted that 5 weekly knockdowns by p120-Kaiso siRNAs since Day 7 not only significantly activated RhoA (Fig. 6A), but also caused sustained JAK2-STAT3 signaling (Fig. 6B–E). Such sustained activation of JAK2-STAT3 signaling as a downstream of RhoA-ROCK was important for the reprogramming of HCECs into NC progenitors as demonstrated by the finding that addition of STAT3 siRNA attenuated BrdU labeling and nuclear translocation of Oct 4, Sox2 and Nanog (Fig. 6G) and blocked overexpression of NC markers (Fig. 6D). Because blockade of sustained activation of STAT3 and its upstream JAK2 also attenuated expression of Bmi1 transcript (Fig. 6B) and nuclear translocation of pBmi1 (Fig. 6E,G), we conclude that JAK2-STAT3 signaling serves as the upstream signaling controlling nuclear translocation of pBmi1, an event critical for downregulating p16^INK4a^. Because STAT3 siRNA did not attenuate nuclear translocation of p120 (Fig. 6F) or Kaiso (not shown) induced by p120-Kaiso siRNAs, nuclear translocation of pBmi1 and nuclear exclusion of p16^INK4a^ by activation of JAK2-STAT3 are not contingent upon p120-Kaiso signaling per se^45^. Collectively, our study has shown that JAK2-STAT3-Bmi1 signaling is another downstream signaling of p120-Kaiso-RhoA-ROCK signaling that participates in reprogramming of HCECs into progenitors via inhibition of p16^INK4a^-mediated senescence.

## Methods

### Materials

Dulbecco’s modified Eagle’s medium (DMEM), advanced DMEM, F-12 medium, bFGF, EGF, phosphate buffered saline (PBS), gentamicin, fetal bovine serum (FBS), knockout serum and Alexa Fluor-conjugated secondary IgG, all real time PCR primers and probes, scrambled (sc) RNA, siRNAs to p120, Kaiso, ROCK1/2, STAT3 and Bmi1, High Capacity Reverse Transcription Kit and CellEvent^TM^ Caspase-3/7 Green Detection Reagent were purchased from Life Technologies (Carlsbad, CA). Insulin-transferrin-sodium selenite (ITS) media supplement and collagenase A was obtained from Roche Applied Science (Indianapolis, IN). LIF, amphotericin B, paraformaldehyde, methanol, Triton X-100, BSA, CT-04 and Hoechst 33342 dye were purchased from Sigma-Aldrich (St Louis, MO). Collagen IV coated 24-well plates were obtained from BD Biosciences (Franklin Lakes, NJ). Polyclonical antibody against p120 was purchased from Santa Cruz Biotechnology Inc (Santa Cruz, CA). Polyclonal antibodies against p16^Ink4a^ (S152), Nanog and Sox2 were obtained from Abcam (La Jolla, CA). Polyclonal pSTAT3 (phospho Y705) antibody was purchased from Cell Signaling (Boston, MA). The monoclonal antibodies against Oct4 and BrdU were purchased from Millipore (Billerica, MA). Monoclonal Bmi1 (F6) antibody was obtained from Life Technologies (Carlsbad, CA). The polyclonal antibody against RhoA and RhoA Activity Assay Biochem kit were obtained from Cytoskeleton (Denver, CO). RNeasy Mini Kit and HiPerFect siRNA transfection reagent were purchased from Qiagen (Valencia, CA).

### HCEC isolation and culture

A total of 64 human corneas from donors aged 24–73 years and maintained at 4 °C in Optisol (Chiron Vision, Irvine, CA) for less than 7 days and the endothelial density of more than 1,500 per mm^2^ were obtained from the Florida Lions Eye Bank (Miami, FL) and handled according to the Declaration of Helsinki. The identity of these cadaver donors cannot be identified. In each experiment, HCEC from the same donor were paired for the control and experimental groups to avoid difference from the donor age. The human corneas were obtained from the Florida Lions Eye Bank (Miami, FL) HCECs were isolated and cultured as previously reported^12^,^46^. In short, after corneal transplantation, the remaining corneoscleral rims were rinsed three times with DMEM containing 50 μg/ml gentamicin and 1.25 μg/ml amphotericin B. Under a dissecting microscope, the trabecular meshwork was cleaned up to the Schwalbe’s line. Descemet’s membranes were stripped from the rim and digested at 37 °C for 16 h with 2 mg/ml collagenase A in modified embryonic stem cell medium (MESCM), which was made of DMEM/F-12 (1:1) supplemented with 10% knockout serum, 10 ng/ml LIF, 4 ng/ml bFGF, 1x ITS supplement, 50 μg/ml gentamicin and 1.25 μg/ml amphotericin B, supplemented with 5% FBS. The resultant clusters of HCECs with undigested basement membrane matrix were collected by centrifugation at 2000 r.p.m for 3 min to remove the digestion solution and cultured in 24-well plates coated with collagen IV in MESCM. Cultures were continuously monitored by phase contrast microscopy.

### siRNA transfection and other treatments

For siRNA knockdown, parallel HCEC monolayers were subjected to 48 hours of transfection at Day 21 or Day 42 or weekly transfection from Day 7 until Day 42 by mixing 50 μl of DMEM with 1 μl of HiPerFect siRNA transfection reagent (final dilution, 1:300) and 1.5 μl of 20 μM scRNA or p120-Kaiso siRNAs, each at the final concentration of 100 nM, drop-wise, followed by culturing in 250 μl of fresh MESCM at 37 °C. Some cultures of HCECs were also added with 5 μg/ml RhoA inhibitor CT-04 or with 100 nM siRNAs to ROCK1/2 or JAK2 or STAT3 or Bmi1 with the last weekly transfection of p120-Kaiso siRNAs in MESCM, depending on the experimental purpose. BrdU was added at a final concentration of 10 μM to a culture 24 h before termination. For each culture, at least 2000 total nuclei were counted for the BrdU labeling index, defined as the number of BrdU-labeled nuclei divided by the total number of labeled and unlabeled nuclei.

### RNA extraction, reverse transcription and real-time PCR

Total RNAs were extracted 24 hours after the last siRNA transfection using RNeasy Mini Kit and reverse-transcribed using High Capacity Reverse Transcription Kit. cDNAs were amplified by real-time RT-PCR using specific primer-probe mixtures and DNA polymerase in 7300 Real Time PCR System (Life Technologies). Real-time RT-PCR profile consisted of 10 min of initial activation at 95 °C, followed by 40 cycles of 15 sec denaturation at 95 °C, and 1 min annealing and extension at 60 °C. The relative gene expression data were analyzed by the comparative CT method (ΔΔCT). All assays were performed in triplicate. The results were normalized by glyceraldehyde 3-phosphate dehydrogenase (GAPDH) as an internal control.

### Immunostaining

The samples were collected 24 hours after the last siRNA transfection, air-dried and fixed in 4% formaldehyde, pH 7.0, for 15 min at room temperature, rehydrated in PBS, incubated with 0.2% Triton X-100 for 15 min, and rinsed 3 times with PBS for 5 min each. For immunostaining to BrdU, samples were fixed with 75% methanol plus 25% acetic acid for 15 min, denatured with 2 M HCl for 30 min at 37 °C and neutralized by 0.1 M borate buffer, pH 8.5 for 5 min 3 times. After incubation with 2% BSA to block non-specific staining for 30 min, the samples were incubated with the desired first antibody (all at 1:50 dilution) for 16 h at 4 °C. After 3 washes with PBS, they were incubated with corresponding Alexa Fluor-conjugated secondary IgG (all 1:100 dilution) for 60 min. The samples were then analyzed with Zeiss LSM 700 confocal microscope (Thornhood, NY). Corresponding mouse and rabbit sera were used as negative controls for primary monoclonal and polyclonal antibodies, respectively.

### β-Galactosidease staining

Senescence β-Galactosidease Staining Kit was obtained from Cell Signaling (Boston, MA). The staining procedure from the manufacturer was followed. In brief, following fixation, β-Galactosidease Staining Solution was added to the sample wells. After incubation at 37 °C in a dry incubator overnight, the β-Galactosidease positive cells were blue-colored under a phase microscope, as shown in dark-black in the photo (Fig. 2) using a black and white phase contrast microscope.

### Western blotting

Cell lysates were prepared in RIPA buffer and resolved on 4–15% (w/v) gradient acrylamide gels under denaturing and reducing conditions for Western blotting. The protein extracts were transferred to a nitrocellulose membrane, which was then blocked with 5% (w/v) fat-free milk in TBST [50 mM Tris-HCl, pH 7.5, 150 mM NaCl, 0.05% (v/v) Tween-20], followed by sequential incubation with the specific primary antibody against keratocan and its respective horseradish peroxidase (HRP)-conjugated secondary antibody using β-actin or α-tubulin as the loading control. Immunoreactive proteins were detected with Western Lighting Chemiluminescence (PerkinElmer, Waltham, MA).

### RhoA activity assay

The assay of Rho activation was performed in 10–50 μg of cell lysate protein using a RhoA Activation Assay Biochem kit to pull down the GTPbound form of RhoA with a GST fusion protein containing rhotekin (7–89 residues) and ras binding domain (RBD) protein using brightly colored glutathione affinity beads. The amount of activated RhoA pulled down was quantitatively determined by Western blotting using anti-RhoA antibody.

### Statistical analysis

All summary data were reported as means ± SD, calculated for each group and compared using ANOVA and the Student’s paired t-test by Microsoft Excel (Microsoft, Redmont, WA). Test results were reported as two-tailed p values, where p < 0.05 was considered statistically significant.

The work is supported by R43 EY022502-01 and R44 EY022502-02 grants from the National Eye Institute, National Institutes of Health, Bethesda, MD, USA (Zhu YT and Tseng SCG), and a research grant from TissueTech, Inc., Miami, FL; and by a grant (81370992) from National Natural Science Foundation of China.

## Additional Information

**How to cite this article**: Lu, W.-J. *et al.* Senescence Mediated by p16^INK4a^ Impedes Reprogramming of Human Corneal Endothelial Cells into Neural Crest Progenitors. *Sci. Rep.* **6**, 35166; doi: 10.1038/srep35166 (2016).
